# Recombinant human erythropoietin increases survival and reduces neuronal apoptosis in a murine model of cerebral malaria

**DOI:** 10.1186/1475-2875-7-3

**Published:** 2008-01-07

**Authors:** Lothar Wiese, Casper Hempel, Milena Penkowa, Nikolai Kirkby, Jørgen AL Kurtzhals

**Affiliations:** 1 Center for Medical Parasitology, University of Copenhagen, Copenhagen, Denmark; 2 Department of Clinical Microbiology, University Hospital Copenhagen, Rigshospitalet, Copenhagen, Denmark; 3 Section of Neuroprotection, The Panum Institute, Faculty of Health Sciences, University of Copenhagen, Copenhagen, Denmark; 4 Department of Zoology, Faculty of Life Sciences, University of Copenhagen, Copenhagen, Denmark

## Abstract

**Background:**

Cerebral malaria (CM) is an acute encephalopathy with increased pro-inflammatory cytokines, sequestration of parasitized erythrocytes and localized ischaemia. In children CM induces cognitive impairment in about 10% of the survivors. Erythropoietin (Epo) has – besides of its well known haematopoietic properties – significant anti-inflammatory, antioxidant and anti-apoptotic effects in various brain disorders. The neurobiological responses to exogenously injected Epo during murine CM were examined.

**Methods:**

Female C57BL/6j mice (4–6 weeks), infected with *Plasmodium berghei *ANKA, were treated with recombinant human Epo (rhEpo; 50–5000 U/kg/OD, i.p.) at different time points. The effect on survival was measured. Brain pathology was investigated by TUNEL (Terminal deoxynucleotidyl transferase (TdT)-mediated deoxyuridine triphosphate (dUTP)-digoxigenin nick end labelling), as a marker of apoptosis. Gene expression in brain tissue was measured by real time PCR.

**Results:**

Treatment with rhEpo increased survival in mice with CM in a dose- and time-dependent manner and reduced apoptotic cell death of neurons as well as the expression of pro-inflammatory cytokines in the brain. This neuroprotective effect appeared to be independent of the haematopoietic effect.

**Conclusion:**

These results and its excellent safety profile in humans makes rhEpo a potential candidate for adjunct treatment of CM.

## Background

*Plasmodium falciparum *malaria remains a massive burden of disease with an estimated two million deaths worldwide each year [1] mainly due to the two major complications, cerebral malaria (CM) and severe anaemia. Moreover long-term neurological sequelae in 2–11% of the survivors of CM are increasingly recognized [2-4]. CM is an acute – and potentially reversible – encephalopathy with increased serum-levels of pro-inflammatory cytokines such as tumor necrosis factor (TNF), interferon-γ (IFN-γ), and lymphotoxin (LT) [5,6]. Post-mortem examination of the brain reveals haemorrhages, oedema and adherence of parasitized red blood cells (pRBC) to the cerebral microvasculature. Although CM has been studied extensively, many of the pathophysiological mechanisms remain unclear and highly effective treatment other than antiparasitic medication is not available. A number of adjunct therapies have been proposed for CM including dexamethasone, hyperimmune serum, anti-TNF antibody, pentoxyfylline, osmotic diuretics, heparin, anti-convulsants and red blood cell (RBC) exchange transfusion, each based on a different pathophysiological rationale. Only anti-convulsants and to a lesser extend RBC exchange transfusion have shown to be partially effective [4].

Erythropoietin (Epo) was first identified as an haematopoietic growth factor produced in the kidneys, and recombinant human Epo (rhEpo) is widely used to treat anaemia [7]. Increased serum Epo levels promote survival of erythroid precursor cells of the bone marrow that would otherwise undergo apoptosis [8]. The observation that Epo and its receptor are expressed in practically all brain cells expanded the biological role of Epo beyond haematopoiesis (reviewed in [9,10]).

In vitro studies have shown that Epo protects neurons against ischaemic damage by reducing apoptosis [11], but the cytoprotective effects are not restricted to neurons: Epo can also protect endothelial cells [12] and glial cells [13]. Apart from its anti-apoptotic effect, Epo may reduced astrocyte activation [14], cause direct neurotrophic effects [15], act as antioxidant [16] and stimulate angiogenesis [17].

Subsequent in vivo studies of systemic treatment with rhEpo reported a reduction of neuronal damage and neurological dysfunction in rodent models after stroke [18-20], mechanical trauma, excitotoxic injury and experimental autoimmune encephalitis (EAE) [21]. Strikingly, a recent study in a mouse model of CM showed an increase of survival in rhEpo-treated animals [22]. Moreover, a proof-of-concept trial in human patients with ischaemic stroke has shown a neuroprotective effect of high-dose rhEpo within six hours after onset of symptoms [23].

Considering these preclinical and clinical data, rhEPO is an attractive potential candidate for the therapy of CM. The aims of the present study were explore the effect of systemic administered rhEpo in an animal model of CM, and thereby to contribute to the body of evidence that can build the basis for a treatment of human CM patients.

Experimental models cannot reproduce all the features of human diseases, and this is true for CM. One major difference from the human disease is that mice do not develop high fevers but instead develop a progressive hypothermia in the days prior to death what is best explained by the big surface area of the mice relative to their size, leading to a heat loss in severe diseased animals. The model used for this study shows however main features also seen in human CM: A clinical picture including coma, seizures and neurological impairment, strong histopathological similarities with impairment of the BBB and petechial bleedings and a systemic as well as a local immune responses having TNF, INF-γ and Il-1β strongly involved as pro-inflammatory cytokines [6,24,25]. The use of rodent malaria parasites in mice is therefore widely accepted as the model of choice for studying the pathogenesis of CM [6,24].

## Methods

### Animals

Female pathogen-free C57BL/6j mice 4–6 weeks old, weighing 18–22 g, were purchased form Taconic, Denmark. A total of 199 mice, 149 for the survival studies was used, 30 for gene expression analysis and 20 for embedding of the brains and staining. The results were obtained from three independent experiments. All animals were pathogen-free and were kept under standard conditions, with *ad libitum *access to food and water. All experiments adhered to Danish and European guidelines for animal research and were approved by the national board for animal studies. All efforts were made to minimize animal suffering and to reduce the number of animals used. The development of ECM is accompanied by a severe drop of body temperature. Mice with a body temperature below 32°C do not recover. Body temperature below 32°C as a proxy for death.

### Induction of ECM

Mice were infected on day 0 with *Plasmodium berghei *ANKA parasites by intraperitoneal inoculation with 10^4 ^parasitized red blood cells (pRBC) from mice of the same strain, diluted in normal saline. Parasitaemia was determined using Giemsa-stained thin blood films. The animals were under daily supervision for clinical signs of disease, body temperature and neurological symptoms. ECM was diagnosed by clinical signs including ataxia, paralysis (mono, hemi, para, or tetraplegia), deviation of the head, convulsions, and coma. Body temperature was measured rectally once or twice a day (Digital thermometer DM852 with rectal probe, Ellab, Denmark).

Mice were treated with erythropoietin alpha, i.p. (Eprex, Janssen-Cilag, Schaffhausen, Switzerland) diluted in NaCl (0.9%) in various dosages (1–200 U daily i.p. diluted in 0,2 ml normal saline per injection) and different treatment schemes. Infected control mice were treated with NaCl (0.9%) only. For immunohistochemistry and rt-PCR analysis of the brains animals were killed on day 8. Survival studies were terminated by killing animals, whith body temperature below 32°C. Surviving animals were killed on day 15 post-inoculation.

### Tissue processing and in situ detection of DNA fragmentation

Tissue processing was performed according to standard procedures, as described elsewhere [26]embedded in paraffin, and cut in serial 5 μm-thick sections. Terminal deoxynucleotidyl transferase (TdT)-mediated deoxyuridine triphosphate (dUTP)-digoxigenin nick end labelling (TUNEL) staining was performed according to manufacturers' protocol using the TdT-FragEL DNA Fragmentation Detection Kit (Oncogene Research Products, Cambridge, UK; code Cat# QIA 33). DAB was used as chromogen and the sections were counterstained with methyl green.

### Visualisation and counts

Sections were examined on an Imager.Z1 microscope with an AxioCam MRc5 camera (Carl Zeiss, Germany). Images were processed using Adobe Photoshop CS (Adobe, USA). In addition to morphological analysis, cellular counting was carried out in a blinded and randomized manner by a single investigator. TUNEL+ cells were counted in sagittal sections from close to the midline of the brain, using one section from each hemisphere.

### Measuring of packed cell volume (PCV)

Tail blood or blood from the retro orbital venous plexus was sampled in EDTA coated capillary tubes (20 μl, Bie and Berntsen, Denmark) and analysed on an automated analyser (KX-21N, Sysmex, USA).

### Gene expression

For quantitative PCR, separate groups of mice (n = 27) were either infected as described above and treated with rhEpo (100 U i.p. on day 1,4 and 7 post-inoculation) or vehicle or left uninfected. Mice were perfused trans-cardially with NaCl (0.9%) with 0.3% heparin (15,000 IU/L) for 30 – 60 seconds. Brains were removed, parted into right and left cerebrum and cerebellum and immediately frozen in liquid nitrogen.

RNA was isolated from homogenized tissue using a standard RNeasy lipid tissue kit (Qiagen, USA) according to the manufacturers' protocol followed by dilution in first Qiazol lysis reagent (Qiagen, USA) and secondly chloroform 99.9% (Sigma, Denmark) to separate phases.

Quantification of RNA content was done by using the Rediplate Ribogreen kit (Invitrogen, USA) and cDNA was synthesized using the QuantiTect Reverse Transcription Kit (Qiagen, USA).

All real time-PCR reactions were carried out on a MX-3000p (Stratagene, USA) and run for 50 cycles using the QuantiTect Probe PCR Kit (Qiagen, USA) according to the manufacturers protocol and the following protein specific kits: β-actin, cat. no. 241014, caspase 3, cat. no. 241124, no. 241054, caspase 1, cat. no. 241151, TNF, cat. no. 241034, IFN-γ, cat. no. 241036, IL-1β, cat. and LT: cat no. 241174. Cycle-threshold (Ct) values were defined as the PCR cycle at which amplified product was first detected. Gene-expression levels were assessed by the comparative threshold method, using expression of the β-actin gene as reference.

### Statistical analysis

Results for multiple groups were evaluated by ANOVA using the Holm-Sidak method for multiple comparisons. Student's-t test were used when comparing two groups and by log-rank statistic for the survival curves with pair wise comparison using the Holm-Sidak method. For rt-PCR data student's-t-test was applied on Ct-values. Cox regression for survival analysis was performed using survival as dependent variable and treatment, parasite level on day 8 and PCV on day 8 as covariates. The heights of the columns represent mean values. Raw data were processed by log- or ln-transformation to improve the homogeneity of variances where necessary. A difference was considered significant for P-values <0.05. Box plot graphs show the percentiles and the median. Cox regression analysis for survival was performed using the SPSS 11.5 software package (SPSS Inc., USA) otherwise were the data analysed using the SigmaPlot 9.01 software package (Systat Software, Inc, USA).

## Results

**Figure 1:**
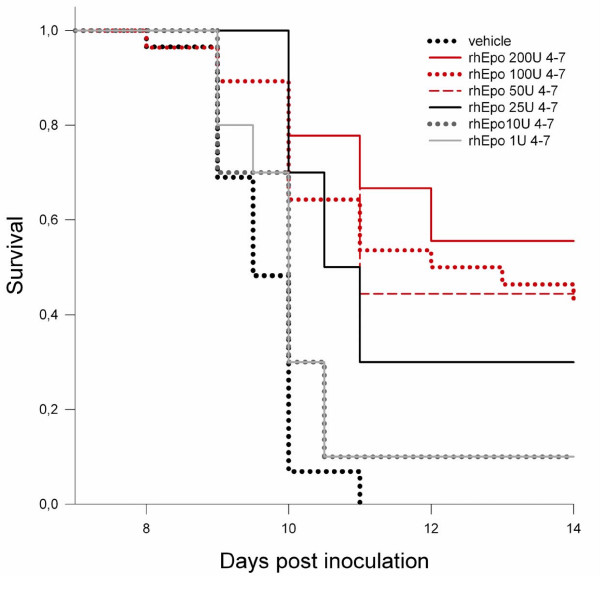
**Dose depandant increase in survival in recombinant human Erythropoietin-treated mice with cerebral malaria**. Dose dependant increase in survival in recombinant human erythropoietin-treated mice with CM: Cumulative survival analysis of mice ECM treated with rhEpo from day 4–7 in different doses from 1–200 U daily versus vehicle treated controls. Mice treated with a high dose (50–200 U daily, red lines) show increased survival by the end of the second week of 44.4%, 42.9% and 55.6% respectively while all saline treated controls (black dotted line) survive no longer than day 11 (p < 0.001). Mice treated with 25 U daily survive in 30% (p < 0.001 vs. vehicle). Survival of mice treated with a low dose (1–10 U daily) did not show a statistically significant increase in survival. Statistical test: log rank statistic for the survival curves with pair wise comparison using the Holm-Sidak method.

Treatment with rhEpo resulted in an increased survival of mice with cerebral malaria in a dose dependent manner. The highest increase in survival was achieved, when mice were treated from day 4 to day 7 post-inoculation with either 200 U, 100 U or 50 U once daily (Figure 1). 56%, 48% and 45% respectively survived until day 14, compared to the saline treated group, where no animal survived longer than day 11 (p <0.001 for the two former groups and p = 0.001 for the latter group, all vs. saline treated controls). No significant difference was seen between the three groups. A lower dose of 25 U rhEpo given daily from day 4–7 resulted in 30% percent survival on day 14 (p = 0.001 vs. saline treated controls), while 1 U and 10 U rhEpo daily did not increase survival significantly.

**Figure 2:**
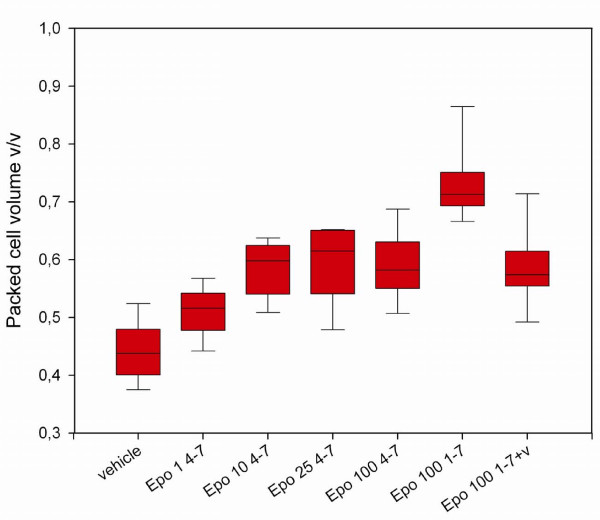
**Packed cell volume in recombinant human erythropoietin-treated and untreated mice with cerebral malaria**. Packed cell volume (PCV) measured on day 8 in animals treated on day 4–7 or 1–7 respectively and vehicle treated controls: Treatment with recombinant human erythropoietin (rhEpo) increases PCV levels in mice with cerebral malaria. 1 U daily from day 4–7 increases the PCV to 51% while 10 U or more daily given on day 4–7 lead to mean PCV levels between 58.4% and 59.7%. PCV levels in the group treated on day 1–7 with 100 U daily reached very high values of 73.0% unless these mice where bled for 10% of their total blood volume on day 5. This intervention reduced the mean PCV level to 58.6% (right plot). P < 0.001. Statistical test: One way ANOVA. The ends of the boxes define the 25th and 75th percentiles, with a line at the median and error bars defining the 10th and 90th percentiles.

RhEpo treatment on day 4–7 increased packed cell volume (PCV) levels measured on day 8 (before killing), compared to saline-treated controls: The lowest dose of 1 U/day of rhEpo increased the PCV from 44.2% (saline-treated, infected controls) to 51.0% and 10 U/day resulted in a PCV level of 58.4%, while higher doses of 25–200 U/day not further increased PCV levels significantly (Figure 2).

**Figure 3:**
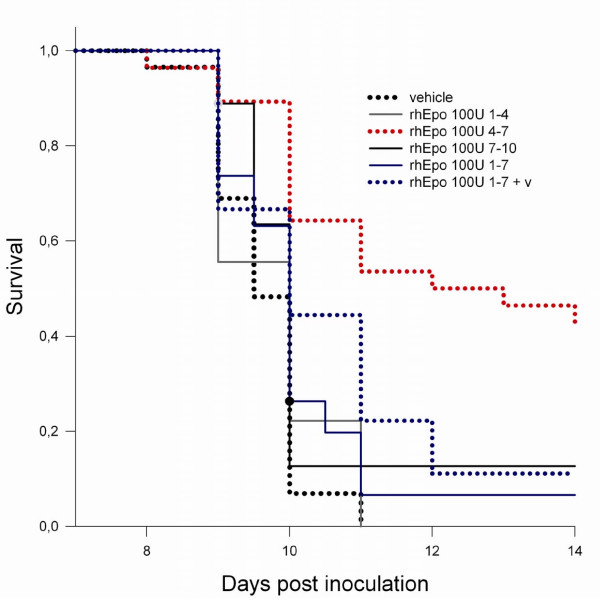
**Time depandant increase in survival in recombinant human erythropoietin-treated mice with cerebral malaria**. Time dependant increase in survival in mice with CM treated with recombinant human Erythropoietin (rhEpo): The cumulative survival analysis of mice ECM treated with 100 U daily of rhEpo show increased survival at the end of the second week only in mice treated from day 4–7 (42.9%, p < 0.001 vs. vehicle). The other treatment schemes used (day 1–4; day 7–10; day 1–7) and mice that received treatment from day 1–7 and where bleed for 10% of their total blood volume to prevent excessively high levels of packed cell volume did not show a statistically significant increase in survival. Statistical test: log rank statistic for the survival curves with pair wise comparison using the Holm-Sidak method.

The effect of the treatment on the time and duration of rhEpo administration: 100 U rhEpo daily was able to increase survival only when administered on day 4–7 (as described above). Early treatment on day 1–4 and late treatment on day 7–10 did not increase survival. Surprisingly, also animals treated for a longer period from day 1–7 with 100 U daily failed to show increased survival. Those animals reached a mean packed cell volume (PCV) of 73% on day 8. In order to eliminate a possible negative effect of the high PCV a group of animals likewise treated on day 1–7 with 100 U rhEpo daily were bled for about 10% of their total blood volume on day 5, resulting in a mean PCV level of 58.6%, which is similar to animals that received treatment only on day 4–7. Despite that, survival was not increased significantly (Figure 3).

**Figure 4:**
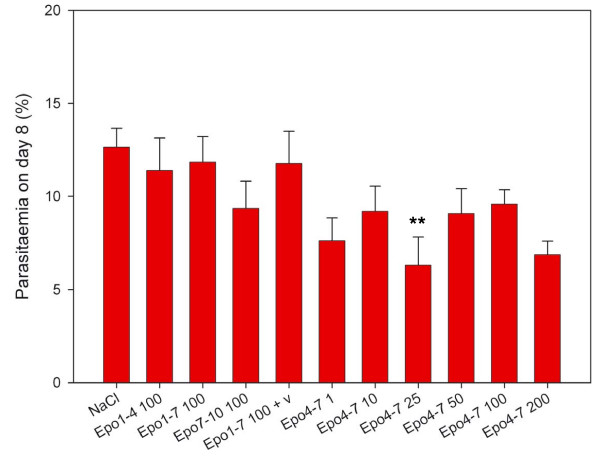
**Mean parasitaemia levels on day 8**. Mean parasiteaemia levels on day 8 as percentage of parasitized red blood cells in Giemsa-stained smears: The mean values for the different groups range from 6.3 – 12.6%. Only the treatment group that received recombinant human Erythropoietin 25 U/day from day 4–7 (**) was significantly different from the control group that had received normal saline (NaCl) (P < 0.05). All other differences between the groups were not significant. Statistical test: One way ANOVA. Data are presented as mean values +/- standard error.

Mean parasitaemia levels on day 8 ranged from 6.3 – 12.6% pRBC (Figure 4). The difference was significant only between animals that had received 25 U/day on day 4–7 and saline treated controls (P < 0.05). The differences between all other groups were not significant. Cox regression analysis to explore the effects of different variables on survival and testing for the covariates treatment, parasitaemia on day 8 and PCV on day 8 showed that only treatment was correlated with increased survival (P < 0.001) while parasitaemia and PCV were not (P = 0.66 and P = 0.74, respectively).

### Body temperature

**Figure 5:**
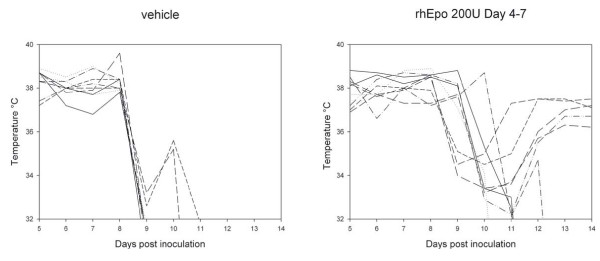
**Body temperature of mice with cerebral malaria**. Vehicle treated mice (left graph) show a characteristic drop of body temperature on day 9. Surviving mice in the group treated with recombinant human Erythropoietin 200 U daily on day 4–7 show a similar drop on day 9–10, but recover afterwards (right graph). The data shown are representative for repeated experiments. The data presented are from one single experiment. Data for the treatment group is representative for mice that received rhEpo 50–200 U daily on day 4–5.

C57BL/6 mice infected with PbA show a characteristic drop in body temperature when entering the terminal phase of disease. Data obtained in a typical experiment are shown in figure 5. The body temperature in the vehicle treated group dropped suddenly, corresponding to the onset of severe clinical disease (coma; paraplegia). The terminal rise of body temperature in two animals coincided with convulsions. Surviving animals in the group treated with rhEpo 200 U daily on day 4–7 had a similar drop in body temperature, but they recovered and reached a stable temperature at the end of the second week (Figure 5). Those animals died later from anaemia and hyperparasitaemia.

### Gene expression

**Figure 6:**
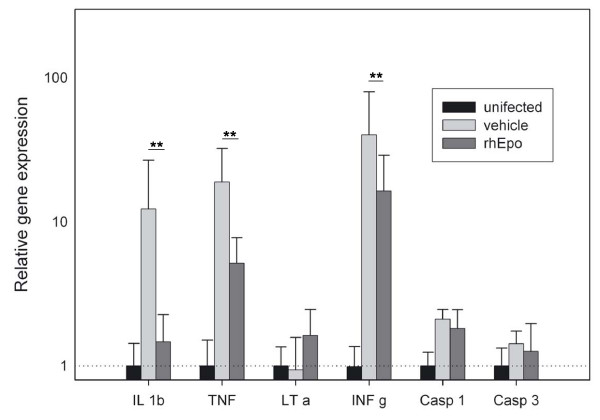
**Gene expression in the brains of recombinant human erythropoietin-treated and untreated mice with cerebral malaria**. Gene expression in the brains of recombinant human erythropoietin-treated and untreated mice with cerebral malaria on day 8 relative to uninfected control mice (black columns). Increased gene expression in infected mice was seen for IL-1β, TNF, INF-γ (P < 0.001) and Caspase 1 (P < 0.05), but not for LT-α, and Caspase 3. Gene expression for the IL-β, TNF and INF-γ was significantly reduced in rhEpo treated mice (P < 0.001). The treatment does not alter the expression of LT-α and the Caspases 1 and 3. Statistical test: Two way ANOVA. Values are presented as mean values relative to uninfected controls. Error bars: Standard deviation (** = P < 0.001).

Gene expression in infected mice was elevated for IL-1β, TNF, INF-γ (P < 0.001) and Caspase 1 (P < 0.05), but not for Caspase 3 and LT, compared to uninfected controls. Treatment with rhEpo was able to decrease this elevation significantly for IL-1β and TNFα (P < 0.001) and for IFN-γ (P = 0.001). The treatment does not alter the expression of LT and the Caspases 1 and 3 (Figure 6).

### Apoptotic neurons

**Figure 7:**
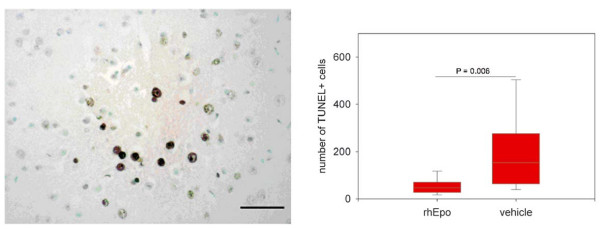
**Treatment with recombinant human erythropoietin in mice with cerebral malaria reduces neuronal apoptosis in the brain**. Apoptotic neurons in the brain of mice with ECM. The micrograph shows TUNEL (Terminal deoxynucleotidyl transferase (TdT)-mediated deoxyuridine triphosphate (dUTP)-digoxigenin nick end labelling) positive nuclei of cells in the cortex of a terminally ill mouse with ECM. Earlier work had shown that the round shaped nuclei belong to neurons [24]. The mean number of TUNEL+ neurons in the brains of recombinant human Erythropoietin (rhEpo) treated mice with ECM is significantly lower than in vehicle treated controls (P = 0.006). Countings from TUNEL-stained sagittal sections of both hemispheres. Scale bar: 50 μm. Statistical analysis: Students t-test. The ends of the boxes define the 25th and 75th percentiles, with a line at the median and error bars defining the 5th and 95th percentiles.

Neuronal apoptosis detected by TUNEL staining is a feature of mice terminal ill with ECM as previously shown [26]. Comparing the number of apoptotic neurons in the brains of rhEpo treated mice and vehicle treated controls with ECM we found a significant decrease in the rhEpo treated group (Figure 7). Furthermore TUNEL+ showed a tendency to cluster (Figure 7). RhEpo treatment reduced the mean number of TUNEL+ neurons counted in sagittal sections from both brain hemispheres form 197.1 (C.I. of the mean: 111.7) in vehicle treated animals to 53.1 (C.I. of the mean: 21.7; P = 0.003).

## Discussion

The data presented here demonstrate a protective effect of rhEpo in ECM in mice with a clear dose and time dependency. animals treated with 50 U to 200 U of rhEpo daily survive in about half of the cases. Treatment with a low dose of 1 or 10 IU daily for the same period of time did not increase survival significantly, while a dose of 25 IU daily increases survival by about 25%. The dose-dependency was seen in mice treated on day 4–7, while early (day 1–4) and late treatments (day 7–10) were unsuccessful. Surprisingly, treatment from day 1–7 failed to increase survival. This failure was in the first place attributed to the very high packed cell volume (PCV) observed in this group (mean: 73% on day 8), as a side effect to the treatment with rhEpo. This could have outweighted a possible positive effect of the treatment. In a subsequent experiment, mice were bled for 150 μl of blood (about 10% of the total blood volume) on day 5 to prevent excessively high values for PCV. This intervention lowered the PCV on day 8 to mean levels equal to those seen in groups responding to treatment, but not with increased survival. A blood loss of 10% of the total volume is substantial and reduces the number of RBC, as well as parasites, thrombocytes and leukocytes, while removing plasma and its contents. However, none of these alterations offer an obvious explanation for the failure of the treatment from day 1–7. An early harmful effect of the treatment cannot be excluded, in line with a study showing that pre-treatment with Epo of C57BL/6 infected with *P*lasmodium *chabaudi *can induced 100% mortality in mice with an otherwise self-limiting course of the disease [27].

The histo-pathological correlate of the neurological dysfunction and sequelae in CM is not known. Axonal injury has been proposed to play a role [28] as well as apoptotic cell death of neurons in ECM in a clustered manner [26]. A recent study by Kaiser *et al *described this pattern only in rhEpo-treated mice, but not in untreated animals with ECM [22]. In our hands both rhEpo-treated mice and untreated mice with ECM develop neuronal apoptosis in this typical clustered manner, while the numbers of TUNEL+ cells as well as the number of clusters in treated animals is reduced (Figure 7). As TUNEL+ neurons are a feature of the very late stage of ECM [26], a possible explanation for this discrepancy between the results presented here and those of Kaiser *et al *could, therefore, be found in the different time points for killing due to different survival times of treated and untreated animals in their study.

Systemic administration of rhEpo has been shown to prevent apoptotic cell death in an animal model of stroke [11], an effect which is likely to be the basis of the reduced infarct size and improved clinical outcome in human stroke patients treated with high dose rhEpo [23]. Accordingly, the present study demonstrated that rhEpo treatment, in addition to increasing survival in mice with ECM, prevents neuronal apoptosis in the CNS. As the Epo-R has been detected on neurons [29], the neuroprotective effect could be due to a direct effect of rhEpo. The data presented here also showed a reduced inflammatory response in the CNS of rhEpo treated mice, as indicated by reduced gene expression of proinflammatory cytokines in the brain tissue; an improved inflammatory response in the brain could contribute to neuroprotection. Further studies are required to determine the sequence of events.

To identify possible pathways leading to apoptosis in ECM, the expression of caspase 3 in the brain was studied. Caspase 3 is involved in signalling pathways leading to apoptosis [30]. It has previously been shown that immunoreactivity for cleaved (i.e. activated) caspase 3 is increased in brains of mice with ECM [26], without a topographical association with TUNEL+ neurons. The gene expression of caspase 3 was not increased in ECM and was independent of rhEpo treatment, indicating that they are possibly not involved in the induction of apoptosis in ECM. However, the induction of apoptosis in ECM might as well have been induced by caspase-independent pathways (reviewed in [30,31]). As caspase 3 is activated by enzymatic cleavage, increased gene expression might not be needed.

The expression of IL-1β, TNF and INF-γ mRNA in the brains increased during ECM, and this increase was reduced by rhEpo treatment (Figure 6). The data for TNF and INF-γ are in line with previous findings [22], and together this current findings for IL-1β clearly indicate that rhEpo-treatment can ameliorate the inflammatory process in ECM. However, the results for LT are surprising: Engwerda *et al *had pointed at the importance of LT, as C57BL/6 mice deficient for LT-α are resistant to CM [32]. In an elegant study, they showed that brain cells are crucial producers of LT-α and proposed endothelial cells, astrocytes or microglia as possible source [32], while Rae *et al *reported a 3–4-fold up-regulation of LT mRNA in the brain of mice with CM [33]. Nevertheless, here LT mRNA levels were not increased in ECM compared to uninfected controls. Adding these findings to the findings of Engwerda *et al*, LT appears to be necessary for the patho-mechanism of ECM, but probably not as a factor triggering cerebral pathology. Treatment with rhEpo had no significant influence on levels of LT expression (P = 0.08), but the power of the test was too low to reject the possibility completely.

The means by which rhEpo treatment may influence the inflammatory response in the CNS remain unclear. It is unlikely that increased levels of PCV are essential, if involved at all, as the induction of erythopoiesis in the different treatment groups did not correlate with increased survival. Even the lowest dose, 1 U daily, had significant erythropoietic effect, and 10 U daily resulted in a maximal induction, not further amplified by higher doses (Figure 1 and 3). By contrast, a dose of 25 U/day rhEpo was necessary to increase survival, with maximal effect when the mice were treated with 50 U to 200 U. The results of the statistical modelling with the Cox regression model clearly support this interpretation, as it detects treatment, but not PCV nor parasitaemia as significantly associated with increased survival. The protective effect of rhEpo in murine CM appears, therefore, to be independent from its haematopoietic effect.

The data on body temperature indicate that the effect of the treatment was via reduced severity as well as delayed course of disease: Surviving animals in the treatment group showed a later, but equal drop in body temperature as vehicle treated mice, before returning to values close to normal (Figure 5). RhEpo treatment in ECM seems to work before, as well as during severe disease. This is of importance as the main target group for a proposed adjunct treatment in CM is patients already having developed signs of severe disease. Marsh et al have previously reported a distinct distribution of CM and severe malaria anaemia (SMA), with respect to age, in children presenting to a hospital in Kilifi, Kenya [34]. Most cases of SMA appear within the first two years of age, while the peak of CM is in the age group 3–4 years. Having these current findings of the neuro-protective effect of Epo and the fact that endogenous Epo levels are high in children with SMA in mind, it can be proposed that these increased levels of endogenous Epo in very young children could contribute to this apparent dichotomy, by protecting against CM.

## Conclusion

RhEpo increased survival in ECM in a dose-dependent manner, was able to reduce the inflammatory response in the brain, and protected functional brain tissue by reducing neuronal apoptosis. It has, therefore, the potential to become a candidate for adjunct treatment of CM in humans, in particular when seen in the light of its excellent safety profile: for decades rhEpo has been used for long term therapy of chronic renal failure. Given in a weekly dose of 50–150 U/kg, it has been shown to be safe for extended periods with rare adverse events (for review see [35]) and experience gained from studies in pre-term infants shows that doses of 2,100 U/week are safe in very young children [36-38]. Furthermore, rhEpo given in a neuroprotective dose of 33,000 IU to stroke patients proved recently to be safe and well-tolerated [23]. Peak plasma concentrations of rhEpo in these patients, as well as in healthy volunteers receiving 2,400 IU/kg rhEpo did not exceed levels of endogenous Epo that can be measured in severely anaemic patients with normal kidney function [7,23,39]. Carefully controlled studies are now indicated and required to proof the concept in humans, including on the timing and the duration of Epo injection.

## Competing interests

The author(s) declare that they have no competing interests.

## Authors' contributions

LW participated in the design of the study, carried out the animal studies and histochemical stainings, performed the statistical analysis and drafted the manuscript. CH and NK performed real-time PCR including data processing. MP participated in the design of the study and the histochemical stainings including microscopy and helped to draft the manuskript. JK participated in the design of the study and helped to draft the manuscript. All authors read and approved the final manuscript.
